# EIF2S1 in Urinary Extracellular Vesicles as a Novel Diagnostic Marker for Bladder Cancer

**DOI:** 10.1002/cam4.70964

**Published:** 2025-05-14

**Authors:** Eisuke Tomiyama, Kazutoshi Fujita, Kyosuke Matsuzaki, Ryohei Narumi, Makoto Matsushita, Yujiro Hayashi, Mamoru Hashimoto, Taigo Kato, Koji Hatano, Atsunari Kawashima, Takafumi Minami, Tetsuya Takao, Shingo Takada, Hirotsugu Uemura, Jun Adachi, Takeshi Tomonaga, Norio Nonomura

**Affiliations:** ^1^ Department of Urology Osaka University Graduate School of Medicine Suita Osaka Japan; ^2^ Department of Urology Kindai University Faculty of Medicine Osaka‐Sayama Osaka Japan; ^3^ Laboratory of Proteomics for Drug Discovery, Center for Drug Design Research National Institutes of Biomedical Innovation, Health and Nutrition Ibaraki Osaka Japan; ^4^ Department of Urology Osaka General Medical Center Osaka Osaka Japan; ^5^ Department of Urology Osaka Police Hospital Osaka Osaka Japan

**Keywords:** bladder cancer, cytoplasmic proteins, extracellular vesicles, liquid biopsy, proteomics, urinary markers

## Abstract

**Background:**

Urinary extracellular vesicles (uEVs), directly secreted from bladder cancer (BCa) cells, harbor potential for biomarker discovery.

**Methods:**

We performed proteomic analysis to explore and validate uEV‐based diagnostic markers for BCa, with a focus on cytoplasmic EV proteins. Among the 1960 proteins identified by shotgun proteomics (tandem mass tag‐labeled liquid chromatography–tandem mass spectrometry [LC–MS/MS]) of uEVs from seven patients with BCa and four healthy individuals, 17 cytoplasmic EV proteins were significantly elevated in the patients' urine (fold change > 1.5; *p* < 0.05). These 17 proteins were subsequently validated using targeted proteomics (selected reaction monitoring/multiple reaction monitoring) using urine samples from 49 and 48 patients with and without BCa, respectively, including those with non‐BCa hematuria.

**Results:**

Ten measurable EV proteins remained significantly elevated in the urine of patients with BCa, with EV‐EIF2S1 demonstrating the best diagnostic performance (area under the receiver operating characteristic [ROC] curve [AUC] [ROCAUC]: 0.83). Additionally, EV‐EIF2S1 distinguished patients with BCa from those without BCa and hematuria in a suitable manner (ROCAUC: 0.92). Functional analysis of EIF2S1 in the BCa cell lines (T24 and 5637) showed that EIF2S1 knockdown markedly inhibited cell proliferation and induced cell cycle arrest and apoptosis, suggesting its essentiality for BCa cell growth and survival.

**Conclusions:**

This study identified EV‐EIF2S1 as a novel, uEV‐based BCa diagnostic marker and demonstrated its functional significance in BCa cell growth and survival.

## Graphical abstract

We conducted a proteomic analysis of urinary extracellular vesicles (uEVs) to identify and validate new diagnostic markers for bladder cancer. Focusing on cytoplasmic EV proteins, we discovered EV‐EIF2S1 as a novel marker and demonstrated its crucial role in the growth and survival of bladder cancer.

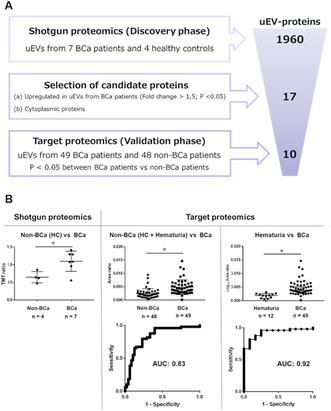

## 1 Introduction

Bladder cancer (BCa) ranks as the second most prevalent urologic malignancy, significantly contributing to global cancer‐related deaths [1]. Although curable through transurethral resection in early stages, BCa prognosis worsens considerably with muscle invasion or metastasis, highlighting the importance of timely diagnosis [2].

Cystoscopy is the primary diagnostic modality for BCa in clinical settings. However, cystoscopy is an invasive procedure, limiting its suitability as a screening test. Nevertheless, many unnecessary cystoscopies are performed to ensure BCa diagnosis is not missed, especially in patients with hematuria. Additionally, repeated cystoscopies during BCa postoperative surveillance impose a significant burden on patients. In contrast, urinary cytology is noninvasive and has high specificity, making it a useful adjunct to cystoscopy. However, its sensitivity is low for low‐grade tumors (approximately 16%) [3], precluding its standalone use as a substitute for cystoscopy [4]. Therefore, developing a noninvasive marker for BCa diagnosis that is superior to urinary cytology remains a critical unmet need.

Extracellular vesicles (EVs) are lipid bilayer vesicles secreted by various cell types that are present in many bodily fluids. EVs contain molecular constituents, such as nucleic acids and proteins, that reflect the characteristics of their origin [5, 6]. Urine contains a high abundance of BCa‐derived EVs owing to its continuous contact with the tumor, making it a promising source of diagnostic markers for BCa [7]. Similar to cells, EVs possess a structure with membrane proteins (receptors and ligands) on the outside and cytoplasmic proteins and RNA within [8]. Our previous study identified upregulated EV proteins in the urine of patients with BCa, presenting them as potential novel diagnostic markers. However, that study focused exclusively on membrane EV proteins [9].

This study aimed to explore and validate urinary EV (uEV)‐based diagnostic markers for BCa, focusing specifically on cytoplasmic EV proteins as a subset of intracellular proteins. Additionally, we investigated the functional role of the identified cytoplasmic protein, EIF2S1, in BCa in vitro.

## 2 Methods

### 2.1 Patients' Urine Sample Collection and Processing

For candidate protein identification through shotgun proteomics (tandem mass tag [TMT]‐labeled liquid chromatography–tandem mass spectrometry [LC–MS/MS]) of urinary EVs (uEVs), urine samples were collected from seven patients with BCa and four healthy individuals at Osaka University Hospital, as previously reported [7, 9]. For the validation of the identified protein candidates, additional urine samples were collected, and their uEVs were analyzed using target proteomics (selected reaction monitoring and multiple reaction monitoring [SRM/MRM]). These samples were obtained from 49 patients with BCa and 48 patients without BCa, from Osaka University Hospital, Osaka General Medical Center, and Osaka Police Hospital, as previously reported [9]. All patients with BCa were histologically diagnosed based on hematoxylin and eosin‐stained sections. Tumors were staged according to the 7th AJCC TNM staging system and graded according to the 2016 World Health Organization criteria. Urine cytology was evaluated according to the Paris system. All patients with non‐malignant hematuria (excluding cystitis cases) underwent cystoscopy, urinary cytology, and computed tomography to rule out malignant diseases, including BCa; as a result, other causes of hematuria, such as kidney stones, were identified. Cystitis cases were clinically diagnosed based on symptoms, identification of the causative bacteria, a favorable response to antibiotic treatment, and negative urinary cytology. The characteristics of the patients analyzed in the shotgun and target proteomics are shown in Table 1. The collected urine samples were kept at 4°C for a maximum of 6 h prior to centrifugation at 2000 × *g* for 30 min. The resulting supernatants were then stored at −80°C until further analysis. The study protocol was approved by the Osaka University Hospital Institutional Review Board (Protocol Number: 13397‐11), and all patients provided written informed consent. All procedures adhered to the Declaration of Helsinki guidelines.

**TABLE 1:** Patient characteristics in shotgun and target proteomics.

	Shotgun proteomics (*n* = 11)		Target proteomics (*n* = 97)		
	Healthy controls (*n* = 4)	Bladder cancer (*n* = 7)	Non‐bladder cancer (*n* = 8)		Bladder cancer (*n* = 49)
			Healthy controls (*n* = 36)	Hematuria (*n* = 12)	
Age (y), median (range)	67.5 (42–79)	73 (66–83)	57 (41–73)	57 (20–82)	71 (31–86)
Sex, *n* (%)					
Male	3 (75.0)	6 (85.7)	22 (61.1)	5 (41.7)	32 (70.0)
Female	1 (25.0)	1 (14.3)	14 (38.9)	7 (58.3)	17 (30.0)
Urine cytology, *n* (%)					
Negative	4 (100)	2 (28.6)	36 (100)	12 (100)	24 (49.0)
Positive	0 (0.0)	5 (71.4)	0 (0.0)	0 (0.0)	24 (49.0)
Unknown	0 (0.0)	0 (0.0)	0 (0.0)	0 (0.0)	1 (2.0)
Pathological T stage *n*, (%)					
pTa	—	3 (42.9)	—		24 (49.0)
pTis	—	0 (0.0)	—		0 (0.0)
pT1	—	0 (0.0)	—		0 (0.0)
pT2 ≤	—	4 (57.1)	—		25 (51.0)
Pathological grade, *n* (%)					
Low‐grade	—	2 (28.6)	—		14 (28.6)
High‐grade	—	5 (7.14)	—		35 (71.4)

### 2.2 Cell Lines and Culture

T24 cells (lot no. 02052018, Apr. 2019) were purchased from the Japanese Collection of Research Bioresources Cell Bank (Osaka, Japan); J82 cells (lot no. 58307736, Oct. 2011), UM‐UC‐3 cells (lot no. 61729357, Aug. 2014), and TCC‐SUP cells (lot no. 57938022, Feb. 2012) were purchased from the American Type Culture Collection (VA, USA); and 5637 cells [lot no. I‐5051 (N6‐2‐7, 43), Aug. 2009] were purchased from the Cell Resource Centre for Biomedical Research, Institute of Development, Aging and Cancer, Tohoku University (Miyagi, Japan). T24, J82, UM‐UC‐3, and TCC‐SUP cells were cultured in Eagle's Minimum Essential Medium (#05975‐56, Nacalai Tesque, Kyoto, Japan), whereas 5637 cells were cultured in Roswell Park Memorial Institute 1640 medium (#30264‐56, Nacalai Tesque, Kyoto, Japan). All media were supplemented with 10% fetal bovine serum (FBS) (Gibco, #10091148), and cells were incubated at 37°C with 5% CO_2_. All cell lines were routinely tested for Mycoplasma contamination using the CycleavePCR Mycoplasma Detection Kit (#CY232, Takara Bio, Shiga, Japan) every 4 weeks. Cells were used for experiments within 8 weeks after thawing, and passage numbers were maintained below 15 during this period.

### 2.3 Isolation of EVs

uEVs were separated using ultracentrifugation with a 30% sucrose/D_2_O cushion, as previously described [10]. The prepared urine samples were centrifuged at 17,000 × *g* for 30 min to eliminate larger EVs. Subsequently, the supernatants were filtered through 0.22‐μm filters and purified by layering on a 30% sucrose/D_2_O cushion followed by ultracentrifugation at 100,000 × *g* for 90 min. Afterward, the sucrose layer was collected, washed twice with phosphate‐buffered saline (PBS), and additionally ultracentrifuged at 100,000 × *g* for 90 min. The final pellet was resuspended in 100 μL of PBS. The isolation of EVs was validated using Western blot, NanoSight particle‐tracking analysis, and transmission electron microscopy, as previously reported [7].

To isolate EVs from cell lines, sub‐confluent cells grown in 15 cm dishes were washed with PBS and then cultured in a medium supplemented with 10% exosome‐depleted FBS for 48 h. The conditioned medium was subsequently collected and centrifuged at 2000 × *g* for 30 min at 4°C to eliminate cell debris, followed by another centrifugation at 17,000 × *g* for 30 min at 4°C to remove large EVs. The supernatant was subsequently filtered through a 0.22‐μm filter, and EVs were isolated using the MagCapture Exosome Isolation Kit PS (#290‐84103, FUJIFILM Wako, Osaka, Japan), according to the manufacturer's instructions [10, 11]. The protein levels of EVs were quantified with a Micro BCA Protein Assay Kit (#23235, Thermo Fisher Scientific, MA, USA).

### 2.4 Selection of Diagnostic Marker Candidates and Peptides for Target Proteomics

From the previously identified 1960 uEV proteins via shotgun proteomics [7], candidates for diagnostic markers were selected based on two criteria: (a) proteins expressed at high levels in uEVs from patients with BCa (fold change > 1.5; *p* < 0.05) and (b) non‐membranous cytoplasmic proteins listed in the UniProt Knowledgebase. To account for sample variation, we normalized EV protein values to CD9 values in each sample. To further confirm the selected candidates as diagnostic markers for BCa using target proteomics, we chose specific peptides according to these criteria: (a) peptides with unique sequences not found in multiple genes; (b) peptides fully digested by trypsin without methionine residues for easier detection; and (c) peptides under 20 amino acids to enhance sensitivity in SRM/MRM assays. Additionally, stable isotope‐labeled internal standard peptides (SI‐peptides), which have the same sequences as the selected peptides but include a C‐terminal 15N‐ and 13C‐labeled arginine or lysine, were acquired from JPT Peptide Technologies GmbH.

### 2.5 Target Proteomics

We employed target proteomics (SRM/MRM analysis) as previously described [7, 9]. Notably, we dissolved digested peptides from 20 μg of EV proteins in a solution containing 2% acetonitrile and 0.1% trifluoroacetic acid. These peptides were then analyzed using a TSQ‐Vantage triple quadrupole mass spectrometer equipped with a nano‐LC interface and other associated equipment. The analytical column used was filled with ReproSil‐Pur C18‐AQ. The mobile phase comprised buffers A and B with varying formic acid and acetonitrile concentrations. The digested peptides, mixed with an SI‐peptide standard, were loaded for LC–MS measurements. A nano‐LC gradient was applied, and the mass spectrometer settings were optimized for SRM/MRM analysis of the target peptides. Collision energy for each peptide was calculated, and data collection was executed in the scheduled SRM mode. Skyline software was used to evaluate the SRM/MRM data by assigning signal peaks to target peptides and comparing them to SI peptide standards. Quantitative values for target peptides were calculated as ratios to isotope‐labeled peptide standards and normalized using CD9 values to account for variability in urinary EV concentrations. For proteins with multiple detectable peptides, the most intense target sequence was used.

### 2.6 SDS‐PAGE and Western Blotting

Cell lysates and EV samples were separated by SDS‐PAGE and transferred onto a polyvinylidene difluoride membrane (#IB24002, Thermo Fisher Scientific, MA, USA) using a dry transfer system (#IB21001S, Thermo Fisher Scientific, MA, USA). The membranes were probed with specific antibodies and subsequently incubated with a horseradish peroxidase‐conjugated secondary antibody against mouse immunoglobulin (1:5000; #7076, Cell Signaling Technology, MA, USA), followed by incubation with enhanced chemiluminescence reagents (#02230, Nacalai Tesque, Kyoto, Japan). Membranes were detected using a ChemiDoc XRS Plus system (#1708265, Bio‐Rad, CA, USA). The following antibodies were used: anti‐CD9 (1:1000; 12A12, Shionogi, Osaka, Japan), anti‐CD63 (1:1000; TS63; Abcam, Cambridge, UK), anti‐EIF2S1 (1:1000; #9722, Cell Signaling Technology, MA, USA), anti‐GAPDH (1:1000; 14C10, Cell Signaling Technology, MA, USA), and anti‐β‐actin (1:1000; #4967, Cell Signaling Technology, MA, USA).

### 2.7 Immunohistochemistry (IHC)

Sections (4‐μm thick) from formalin‐fixed, paraffin‐embedded BCa tissue were deparaffinized using xylene and alcohol. Antigen retrieval was performed using 10 mM citrate buffer (pH 6.0) and microwave treatment for 10 min. Endogenous peroxidase activity was further blocked by incubating with 0.3% hydrogen peroxide for 5 min, followed by overnight incubation with a primary antibody against EIF2S1 (1:250; AHO0802, Thermo Fisher Scientific, MA, USA) at 4°C. Subsequently, we used the EnVision+ detection system for signal amplification (#K4065, DAKO, Glostrup, Denmark) according to the manufacturer's instructions. The sections were counterstained with hematoxylin, dehydrated using a graded series of ethanol concentrations, cleared in xylene, and mounted for microscopic observation. IHC score was evaluated by the percentage of stained tumor cells and staining intensity and was scored from 0 to 3+ (0: no staining, 1+: weak, 2+: moderate, and 3+: strong). The average IHC score of the three randomly selected fields (400×) was calculated in each case as the final result.

### 2.8 RNA Interference

Human BCa cell lines T24 and 5637 were transfected with 10 nM of EIF2S1‐targeting siRNA (#1027416, Qiagen, Hilden, Germany) or a control siRNA (#1027281, Qiagen, Hilden, Germany) using Lipofectamine RNAiMAX Transfection Reagent (#13778075, Thermo Fisher Scientific, MA, USA). Twenty‐four hours post‐transfection, the medium containing siRNA and transfection reagent was replaced with a fresh maintenance medium. Subsequent functional assays were conducted following confirmation of EIF2S1 knockdown via Western blotting.

### 2.9 Establishment of EIF2S1‐Overexpressing BCa Cell Lines

Subconfluent BCa cell lines (T24 and J82) were transfected in 6‐well plates using 3 μL of Lipofectamine 2000 reagent (Thermo Fisher Scientific, MA, USA) and 1 μg of EIF2S1‐coding plasmids or control plasmids (pcDNA3.1 + C‐DYK plasmid, GenScript, NJ, USA). Twenty‐four hours post‐transfection, the media containing plasmids and transfection reagent were replaced with maintenance media. Two days after transfection, the cells were subjected to selection with 0.6 mg/mL G418 for 2 weeks. EIF2S1 overexpression was confirmed by Western blotting.

### 2.10 MTS Cell Proliferation Assay

T24 and 5637 cells transfected with EIF2S1 or a negative control siRNA were plated in 96‐well plates (2.0 × 10^3^ cells/well) and incubated at 37°C for 24 h, with 5% CO_2_ for the indicated time. In total, 20 μL of Cell Titer 96 AQueous One Solution Reagent (#G3580, Promega, WI, USA) was added, and the plates were incubated again at 37°C and 5% CO_2_ for 90 min. The absorbance was measured at 490 nm using a microplate reader (#1681130, Bio‐Rad, Hercules, CA, USA). The assay was repeated three times for each experimental group.

### 2.11 Cell Cycle Assay

Deep Red Cell Cycle Assay Solution (#C548, Dojindo, Kumamoto, Japan) was used to measure the cell cycle according to the manufacturer's instructions. Twenty‐four hours after transfection with siRNA, the treated T24 cells (5 × 10^5^ cells) were collected and washed twice with PBS. The cells were subsequently incubated with 5 μL of Cell Cycle Assay Solution for 15 min at 37°C in the dark, and each sample was filtered through a cell strainer. Sample analysis was performed on a flow cytometer using APC‐Cy7 to determine cell cycle distribution, and the percentage of cells in each phase (G0/G1, S, and G2/M) was calculated using FlowJo Software (version 10.10.0, FlowJo LLC, OR, USA).

### 2.12 TUNEL Apoptosis Assay

Apoptosis was assessed using the In Situ Apoptosis Detection Kit (#MK500, Takara Bio, Shiga, Japan) following the TUNEL (TdT‐mediated dUTP nick‐end labeling) method, according to the manufacturer's instructions. Twenty‐four hours after siRNA transfection, T24 cells (1 × 10^5^ cells/well) were seeded onto 2‐well chamber slides and incubated overnight. The cells were then washed with PBS, fixed with 4% paraformaldehyde for 15 min, and washed again with PBS. Subsequently, the permeabilization buffer was applied for 5 min on ice, followed by another PBS wash. For TUNEL labeling, 50 μL of the reaction mixture (comprising 5 μL TdT Enzyme and 45 μL Labeling Safe Buffer, prepared and cooled on ice before use) was added to each slide. Slides were incubated in a humidified chamber at 37°C for 90 min and then washed with PBS. Finally, the cells were analyzed using a fluorescence microscope. TUNEL‐positive cells and total cell numbers were counted using ImageJ [12], and the percentage of TUNEL‐positive cells was calculated.

### 2.13 Survival Analysis

The association between EIF2S1 expression levels and overall survival was evaluated in patients with BCa from the TCGA cohort (*n* = 413) [13]. Patients were divided into two groups (high‐expression and low‐expression) based on EIF2S1 expression levels, and survival curves were compared between the groups.

### 2.14 Data Analysis and Statistics

The selected proteins after shotgun proteomics were analyzed using DAVID tools (https://david.ncifcrf.gov/tools.jsp) for KEGG Pathway Enrichment analysis [14]. Statistical analyses were performed using JMP Pro (version 16.0.0, SAS Institute, NC, USA), and measurements were quantified with GraphPad Prism (version 7.05, GraphPad Software, CA, USA). Univariate analysis was conducted using Welch's *t*‐test, two‐tailed Student's *t*‐test, or the Mann–Whitney *U* test, as appropriate. Survival rates were analyzed using the Kaplan–Meier method, and comparisons were made using the log‐rank test. *p*‐Values less than 0.05 were considered statistically significant. Diagnostic performance was evaluated using the area under the receiver operating characteristic (ROC) curve (AUC). The optimal diagnostic threshold was determined using the Youden index, and sensitivity and specificity were calculated based on the identified cutoff value.

## 3 Results

### 3.1 Proteomic Identification of EIF2S1 as a BCa Diagnostic Marker

**FIGURE 1:**
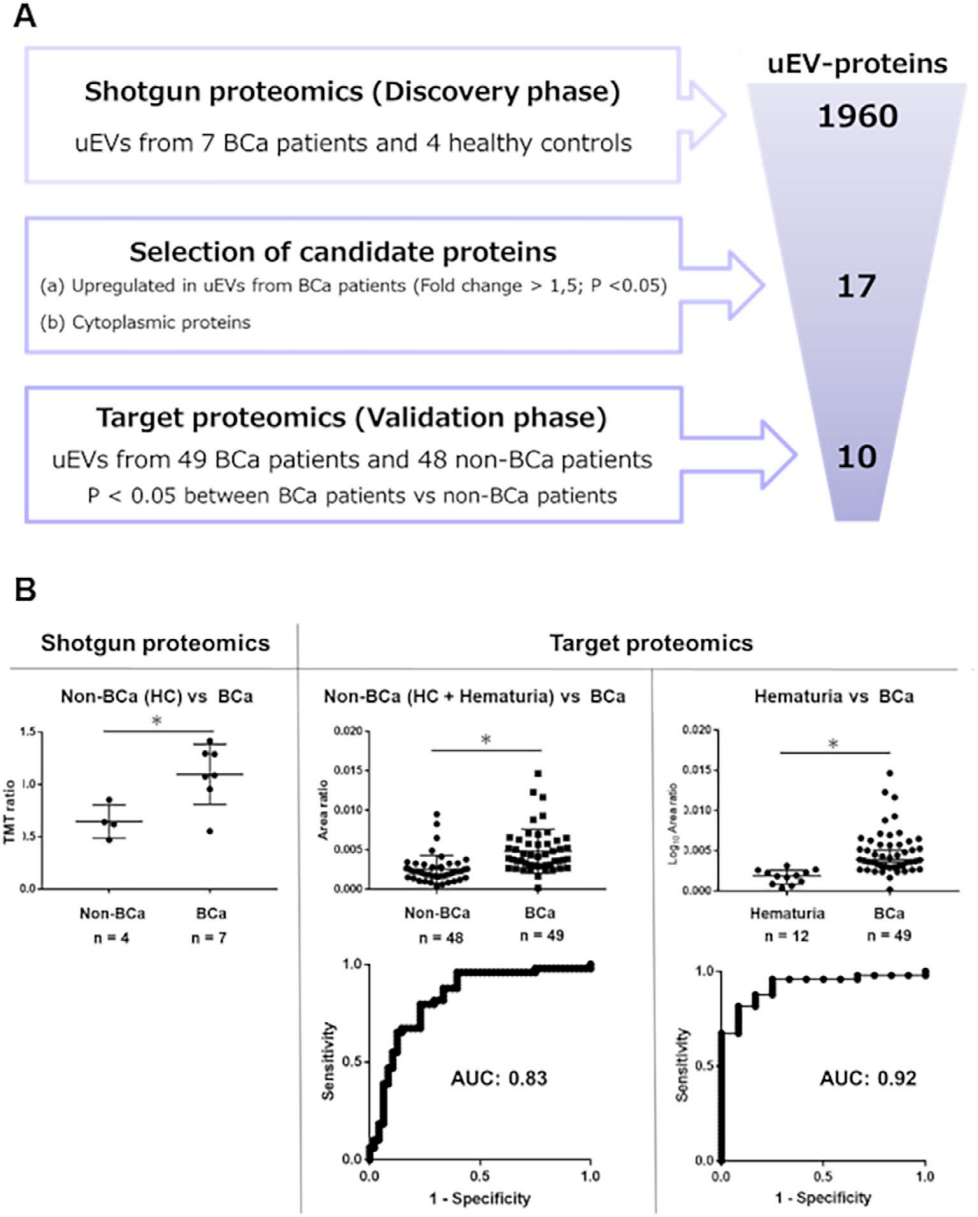
(A) Summary of the identification process for diagnostic markers of bladder cancer (BCa) in urinary extracellular vesicles (uEVs). The selection criteria and the number of candidate proteins shortlisted at each step are detailed. (B) Comparison of relative quantification values for uEV‐EIF2S1 between patients with and without BCa, analyzed in shotgun and targeted proteomics (top). The diagnostic performance of uEV‐EIF2S1 for BCa was assessed using receiver operating characteristic (ROC) curve analysis, with the area under the curve (AUC) values provided (bottom). Data are shown as medians with 95% confidence intervals (Mann–Whitney *U* test; ***p < 0.01).

The workflow for identifying candidate uEV proteins for diagnosing BCa is shown in Figure 1A. Of the 1960 uEV proteins previously discovered through shotgun proteomics in seven patients with BCa and four healthy controls [7], we selected 17 cytoplasmic uEV proteins that were significantly upregulated in patients with BCa (fold change > 1.5; *p* < 0.05; Welch's *t*‐test) (Table S1). We performed KEGG Pathway Enrichment analysis with these 17 proteins, revealing that pathways related to glycolysis/gluconeogenesis and mitophagy/autophagy are significantly enriched (Figure S1).

To validate the selected cytoplasmic uEV proteins as BCa diagnostic markers, we performed target proteomics (SRM/MRM) on uEVs from a cohort of 97 individuals, comprised of 49 patients with BCa and 48 without, including 36 healthy individuals and 12 with nonmalignant hematuria or cystitis. Among the 17 candidate cytoplasmic uEV proteins, 10 were detectable via SRM/MRM and validated to be significantly upregulated in patients with BCa compared to those without BCa (*p* < 0.05; Mann–Whitney *U* test) (Table 2). Notably, eukaryotic translation initiation factor 2 subunit alpha (EIF2S1) demonstrated the highest diagnostic capability (ROCAUC: 0.83, sensitivity: 77.6%, specificity: 72.9%), surpassing urine cytology's sensitivity and specificity (49.0% and 100%, respectively). In addition, EIF2S1 showed a significantly higher expression in the uEVs from patients with BCa (*n* = 49) than from patients with hematuria (*n* = 12) (ROCAUC: 0.92, sensitivity: 82%, and specificity: 83%). We also performed a multigroup analysis by dividing the BCa group by tumor grade [low‐grade (*n* = 14) and high‐grade (*n* = 35)] and found that patients with low‐grade and high‐grade BCa had significantly higher uEV‐EIF2S1 expression compared to individuals without BCa (Bonferroni‐corrected Mann–Whitney *U* test, Figure S2A). The diagnostic performance of uEV‐EIF2S1 for low‐grade BCa showed an ROCAUC of 0.77, with a sensitivity of 85.7% and a specificity of 66.7%. Additionally, we analyzed the data separately for male and female patients and observed significant differences between the non‐BCa and BCa groups in both sexes, indicating that the diagnostic utility of uEV‐EIF2S1 is not significantly influenced by sex (Mann–Whitney test, Figure S2B). The relative quantification values of uEV‐EIF2S1 from proteomic analyses (shotgun and target proteomics) are summarized in Figure 1B, and the corresponding data for the remaining nine uEV proteins are presented in Figure S3. The list and corresponding levels of the 14 peptides associated with the 10 proteins analyzed in the targeted proteomics are shown in Tables S2 and S3, respectively.

**TABLE 2:** Results of proteomic analysis of 10 candidate uEV‐proteins validated in target proteomics.

Protein name	Shotgun proteomics (*n* = 11)		Target proteomics (*n* = 97)					
	BCa (*n* = 4) versus HC (*n* = 7)		BCa (*n* = 49) versus non‐BCa (HC+ hematuria) (*n* = 48)			BCa (*n* = 49) versus hematuria (*n* = 12)		
	Fold change	*p*	Fold change	*p*	AUC	Fold change	*p*	AUC
① Eukaryotic translation initiation factor 2 subunit alpha (EIF2S1)	1.6974	0.0084	1.8995	0.0001	0.8295	2.6909	0.0001	0.9235
② Transketolase (TKT)	1.7826	0.0356	2.8265	0.0001	0.7925	1.8567	0.3408	0.5901
③ Neuroblast Differentiation‐Associated Protein AHNAK (AHNK)	1.6158	0.0444	2.5970	0.0001	0.7730	1.8350	0.1765	0.6276
④ 14‐3‐3 protein zeta/delta (1433Z)	1.5584	0.0485	1.7737	0.0001	0.7674	1.3355	0.2968	0.5986
⑤ 14‐3‐3 protein theta (1433T)	1.7807	0.0445	1.6031	0.0001	0.7594	1.3850	0.1084	0.6514
⑥ Calreticulin (CALR)	4.7156	0.0288	4.3392	0.0001	0.7479	3.8640	0.0250	0.7109
⑦ Cystatin‐B (CYTB)	1.7292	0.0192	2.1562	0.0001	0.7245	1.2879	0.8918	0.5136
⑧ Eukaryotic translation initiation factor 4H (EIF4H)	2.9069	0.0204	1.5162	0.0002	0.7215	1.3627	0.1492	0.6361
⑨ Protein S100‐P (S100P)	2.0524	0.0403	2.4673	0.0003	0.7126	1.5127	0.7099	0.5357
⑩ Y‐box‐binding protein 1 (YBOX1)	N/C	0.0168	2.1275	0.0008	0.6969	1.6693	0.1946	0.6225

Abbreviations: BCa, bladder cancer; HC, healthy controls; N/C, not calculated due to no expression in HC; uEV, urinary extracellular vesicles.

### 3.2 EIF2S1 Expression in EVs Derived From BCa Cell Lines and Tissues

**FIGURE 2:**
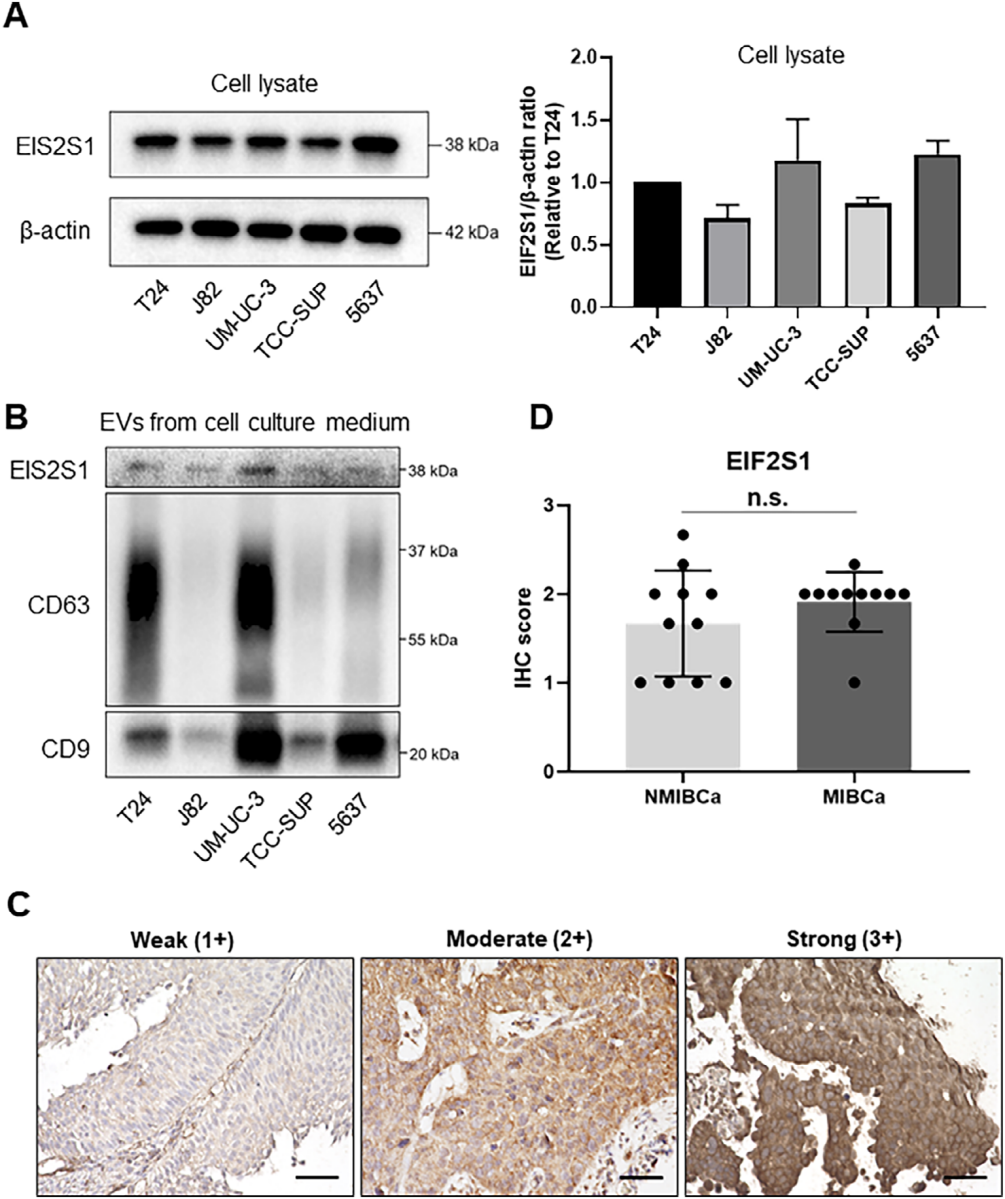
(A) Western blotting and quantification of EIF2S1 expression in five bladder cancer (BCa) cell lines. Data are presented as the mean with SD (*n* = 3). (B) Western blotting of EIF2S1 expression in extracellular vesicles (EVs) extracted from the conditioned media of five BCa cell lines. CD9 and CD63 were used as marker proteins of EVs. (C) Representative immunohistochemical staining of EIF2S1 in BCa tissues (pTa and pT2). Scale bars: 50 μm. (D) Comparison of IHC scores of EIF2S1 between pTa tumors (*n* = 11) and pT2 tumors (*n* = 11). Data are presented as mean with SD. n.s., not significant.

We examined the expression of EIF2S1 in five BCa cell lines using Western blotting. Among the five BCa cell lines, T24, UM‐UC‐3, and 5637 exhibited the highest EIF2S1 expression levels (Figure 2A and Figure S4A). Additionally, we performed Western blotting to confirm EIF2S1 expression in EVs derived from BCa cells, and EIF2S1 expression was detected in the EVs of each cell line (Figure 2B and Figure S4B). We further conducted IHC analysis to evaluate EIF2S1 expression in BCa tissue samples [NMIBCa (*n* = 11) and MIBCa (*n* = 11)]. The clinical and pathological characteristics of the patients are summarized in Table S4. Our findings revealed that EIF2S1 was expressed in BCa tissues (Figure 2C), with no significant difference in IHC scores between NMIBCa and MIBCa samples (*p* = 0.272, Mann–Whitney *U* test, Figure 2D). The presence of EIF2S1 in BCa tissues, irrespective of NMIBCa or MIBCa status, suggests that these tissues could be a potential source of EIF2S1 in urinary EVs from patients with BCa.

### 3.3 Knockdown of EIF2S1 Attenuates Cell Growth and Induces Apoptosis of BCa Cells

**FIGURE 3:**
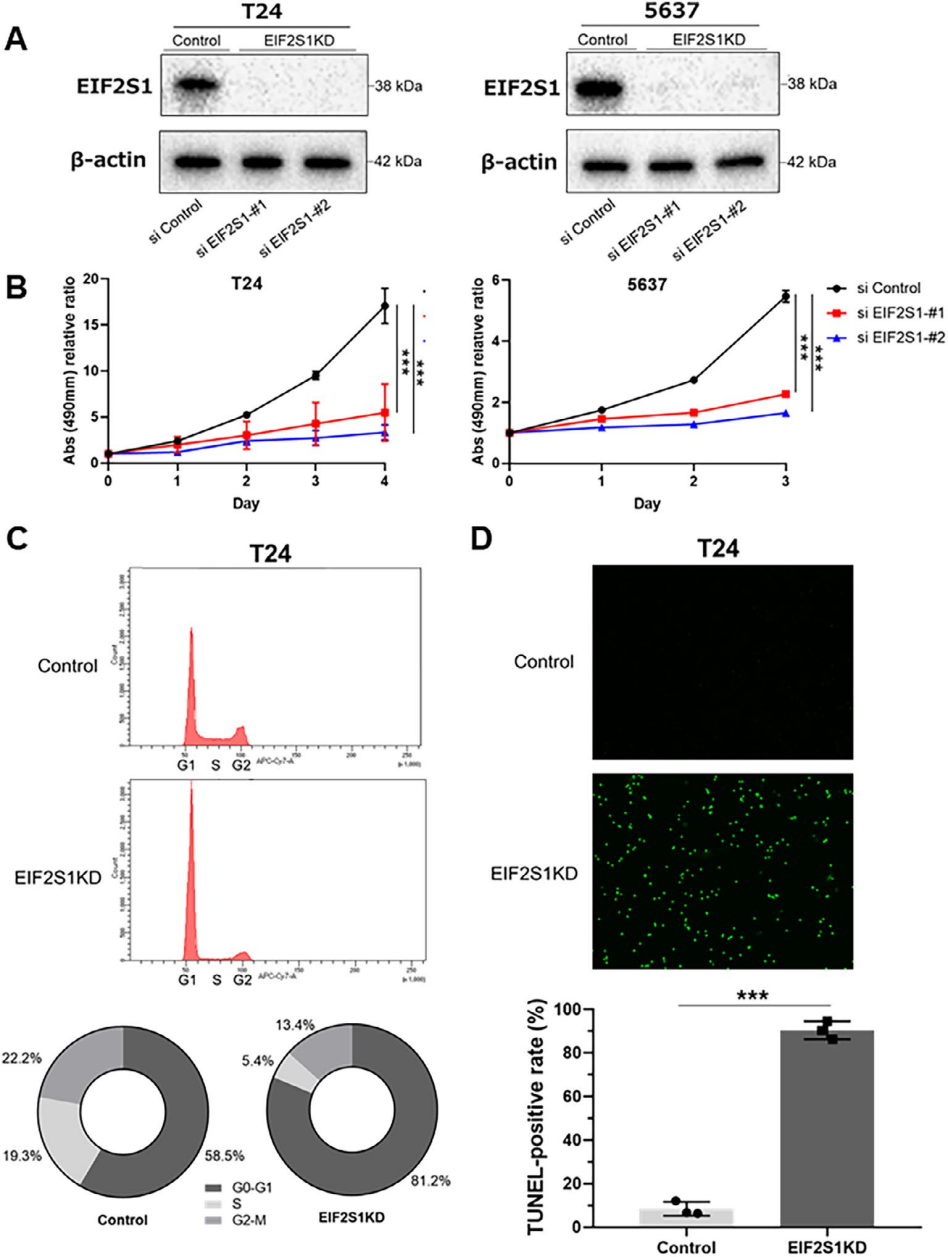
(A) Western blotting showing EIF2S1 was knocked down (KD) with siRNA in the lysate of T24 and 5637 cells. (B) T24 and 5637 cells were transfected with siRNA for 24 h and then incubated for the indicated time periods. Cell proliferation was assessed using the MTS assay. Results are presented as mean with SD (*n* = 3) and analyzed using the Student's *t*‐test (***p < 0.01). (C) Cell cycle assay showing KD of EIF2S1 prevented T24 cells from progressing from G1 to stages S and beyond. The pie chart represents the percentage of cells in each phase (G0/G1, S, and G2/M). (D) Representative images and quantification of TdT‐mediated dUTP nick‐end Labeling (TUNEL) assay showing KD of EIF2S1‐induced apoptosis in T24 cells. Data are presented as mean with SD (*n* = 3) and analyzed using the Student's *t*‐test (***p < 0.01).

To explore the biological role of EIF2S1 in BCa cells, we conducted siRNA‐mediated knockdown experiments in T24 and 5637 cell lines. We confirmed the downregulation of EIF2S1 by the two siRNAs using Western blotting. The knockdown significantly suppressed the proliferation of both T24 and 5637 cells (*p* < 0.05) (Figure 3A,B, and Figure S4C).

Furthermore, we evaluated the effect of EIF2S1 knockdown on BCa cell cycle progression. The cell cycle profiles were determined by staining the DNA with a fluorescent dye and measuring its intensity, which demonstrated that the knockdown of EIF2S1 prevented the progression of BCa (T24) cells from G1 to S and beyond (Figure 3C). In addition, we conducted a TUNEL apoptosis assay and found that the knockdown of EIF2S1 induced apoptosis in T24 cells (Figure 3D).

Additionally, the prognostic impact of EIF2S1 expression on the survival of patients with BCa in the TCGA cohort (*n* = 408) was assessed. Kaplan–Meier survival analysis showed that patients with low EIF2S1 expression had significantly poorer overall survival compared to those with high expression (*p* = 0.008, log‐rank test; Figure S5). These findings collectively suggest that EIF2S1 plays a crucial role in BCa cell growth and survival. However, despite its essential role, EIF2S1 overexpression did not enhance the proliferation of either T24 or J82 cells (Figure S6). This indicates that EIF2S1 alone is insufficient to drive BCa cell proliferation.

## 4 Discussion

The direct contact between urine and BCa makes it a promising source for noninvasive BCa diagnostics [15]. However, extracting cancer‐specific information from urine is often complicated owing to the presence of various contaminants. Cancer‐derived EVs, encapsulating molecules mirroring the characteristics of cancer cells, are gaining significant attention as potential sources of novel diagnostic markers for various cancers [5, 6]. Notably, urine harbors abundant BCa‐derived EVs, enabling the identification of cancer‐specific molecules. Among these, proteins expected to play functional roles hold substantial promise as noninvasive diagnostic markers and as potential therapeutic targets [7].

Although several reports have identified and validated uEV proteins as diagnostic markers for BCa [7, 9, 16, 17, 18], these primarily focused on membrane EV proteins for analysis in immune assays, such as enzyme‐linked immunosorbent assay and fluorescence‐activated cell sorting, neglecting intracellular EV proteins. Although membrane proteins are attractive because of their detectability in immune assays, cytoplasmic EV proteins are also emerging as promising diagnostic markers for BCa. With advancements in mass spectrometry, the quantification of cytoplasmic proteins is now feasible. Additionally, cytoplasmic proteins play crucial intracellular roles, suggesting that focusing on the cytoplasmic proteins encapsulated in EVs may reveal biomarkers with functional significance in cancer.

Our study focused on cytoplasmic EV proteins and identified EV‐EIF2S1 as a novel diagnostic marker for BCa that exhibits superior diagnostic performance compared with urinary cytology. Furthermore, its solid performance (sensitivity: 82%, specificity: 83%) in a cohort that included patients with hematuria highlights its potential as a reliable diagnostic tool for BCa in a clinical hematuria setting.

Notably, the pathway enrichment analysis revealed that uEV proteins from patients with BCa are significantly enriched in the glycolysis/gluconeogenesis and mitophagy/autophagy pathways, which are known to be upregulated in cancer cells to maintain high metabolic activity and survival under stressful conditions [19, 20], suggesting that uEVs are potential biomarkers for BCa diagnosis and provide valuable insight into the metabolic adaptations and survival strategies of BCa cells.

Our functional analysis demonstrated that EIF2S1 knockdown in BCa cells induces cell cycle arrest and subsequent apoptosis. Given that EIF2S1 plays a crucial role in initiating protein synthesis and regulating cellular stress responses [21, 22], its downregulation is expected to impair protein synthesis, affecting key cell cycle regulators such as cyclins, thereby leading to G1/S phase arrest [23]. Moreover, EIF2S1 knockdown reportedly disrupts stress response pathways, including the unfolded protein response and integrated stress response, resulting in reduced ATF4 expression, which exerts a protective effect against cell death by inducing adaptive autophagy [24]. In several cancer types, increased expression levels of EIF2S1 are associated with more aggressive types of cancer [21]. For example, EIF2S1 knockdown suppressed cell invasion/migration in liver cancer and correlated with poor prognosis in hepatocellular carcinoma [25]. In addition, EIF2S1 has been identified as a promising therapeutic target in intestinal‐type adenocarcinoma [26]. In contrast, in squamous cell carcinoma, inhibition of EIF2S1 is compensated for by an alternative translation initiation factor, EIF2A, thereby sustaining the survival of squamous cell carcinoma despite the inhibition of EIF2S1 [27]. Therefore, the dependence on EIF2S1 varies among cell types. However, given that EIF2S1 promotes the survival and progression of certain cancer cells and is associated with poor prognosis in BCa, targeting EIF2S1 may be a potential therapeutic option in BCa management.

This study had some limitations. First, owing to the small cohort size, the utility of urinary EV‐EIF2S1 as a BCa marker should be validated in a larger cohort. Second, we did not include patients with other urological cancers, such as prostate cancer and renal cell carcinoma, which could potentially influence urinary EV profiles. Further investigation is needed to evaluate whether concomitant urological diseases could influence the amount of EIF2S1 in uEVs. Third, the role of EIF2S1 as an EV protein requires further investigation. Although this study elucidated the importance of EIF2S1 in BCa, a potential future approach could be to collect EVs from EIF2S1‐overexpressing cells and incubate them with wild‐type or EIF2S1 low‐expressing cells to assess the functional impact of EIF2S1 as an EV protein.

In conclusion, our proteomic analysis of uEVs identified the cytoplasmic EV protein EIF2S1 as a novel diagnostic marker for BCa. This EV protein demonstrates robust diagnostic capabilities, even in the presence of hematuria, suggesting its potential as a valuable tool for BCa diagnosis. Although this study focuses on the diagnostic potential of uEV‐EIF2S1, longitudinal measurements of this marker could provide valuable insights into its role in monitoring disease progression in BCa. Additionally, the observed functional impact of EIF2S1 in BCa cells highlights its potential as a therapeutic target, warranting further investigation in future studies.

## Author Contributions

**Eisuke Tomiyama:** conceptualization (equal), data curation (equal), formal analysis (equal), funding acquisition (equal), investigation (equal), resources (equal), validation (equal), writing – original draft (equal). **Kazutoshi Fujita:** conceptualization (equal), funding acquisition (equal), supervision (equal), writing – review and editing (equal). **Kyosuke Matsuzaki:** data curation (equal), formal analysis (equal), funding acquisition (equal), investigation (supporting). **Ryohei Narumi:** data curation (equal), formal analysis (equal), methodology (equal). **Makoto Matsushita:** formal analysis (supporting), investigation (supporting). **Yujiro Hayashi:** formal analysis (supporting), investigation (supporting). **Mamoru Hashimoto:** resources (equal). **Taigo Kato:** writing – review and editing (equal). **Koji Hatano:** writing – review and editing (equal). **Atsunari Kawashima:** writing – review and editing (equal). **Takafumi Minami:** resources (equal). **Tetsuya Takao:** resources (supporting). **Shingo Takada:** resources (supporting). **Hirotsugu Uemura:** writing – review and editing (equal). **Jun Adachi:** data curation (equal), formal analysis (equal), methodology (equal), supervision (equal). **Takeshi Tomonaga:** methodology (equal), supervision (equal). **Norio Nonomura:** project administration (equal), supervision (equal), writing – review and editing (equal).

## Ethics Statement

The study protocol was approved by the Osaka University Hospital Institutional Review Board (Protocol Number: 13397‐11), and all patients provided written informed consent. All procedures adhered to the Declaration of Helsinki guidelines.

## Conflicts of Interest

The authors declare no conflicts of interest.

## Supporting information


Tables S1–S4.

Figures S1–S6.

## Data Availability

The datasets generated and analyzed during this study are available from the corresponding author upon reasonable request.
