# Supplementary material for: EIF2S1 in Urinary Extracellular Vesicles as a Novel Diagnostic Marker for Bladder Cancer
Source: Cancer Med. 2025 May 14;14(10):e70964. doi: 10.1002/cam4.70964 (PMC12076193; doi:10.1002/cam4.70964)
Supplement: Supplementary file 2 — Figures S1–S6. [file CAM4-14-e70964-s002.docx]

**Supplementary Figures**

**Figure S1**

**Figure S1**

The top 10 enriched KEGG pathways of 17 selected proteins after shotgun proteomic analysis.

**Figure S2**


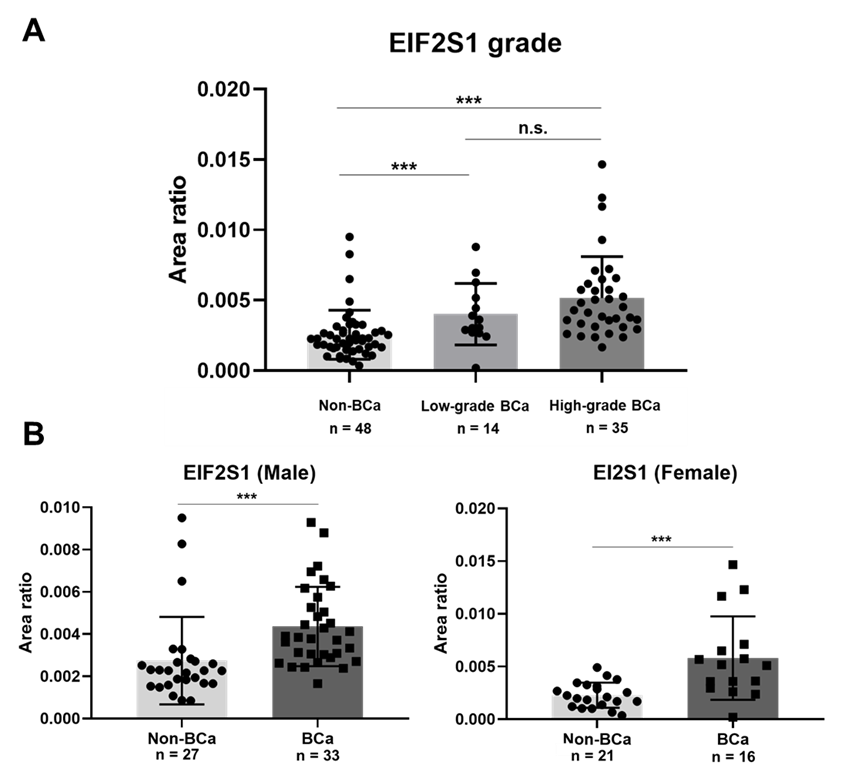


**Figure S2**

Comparison of quantification values of uEV-EIF2S1 in target proteomics (A) across non-BCa, low-grade BCa, and high-grade BCa groups (Bonferroni-corrected Mann–Whitney U test) and (B) between non-BCa and BCa groups, separated by sex (Mann–Whitney U test). ***p < 0.01. uEV, urinary extracellular vesicles; BCa, bladder cancer.

**Figure S3**

**
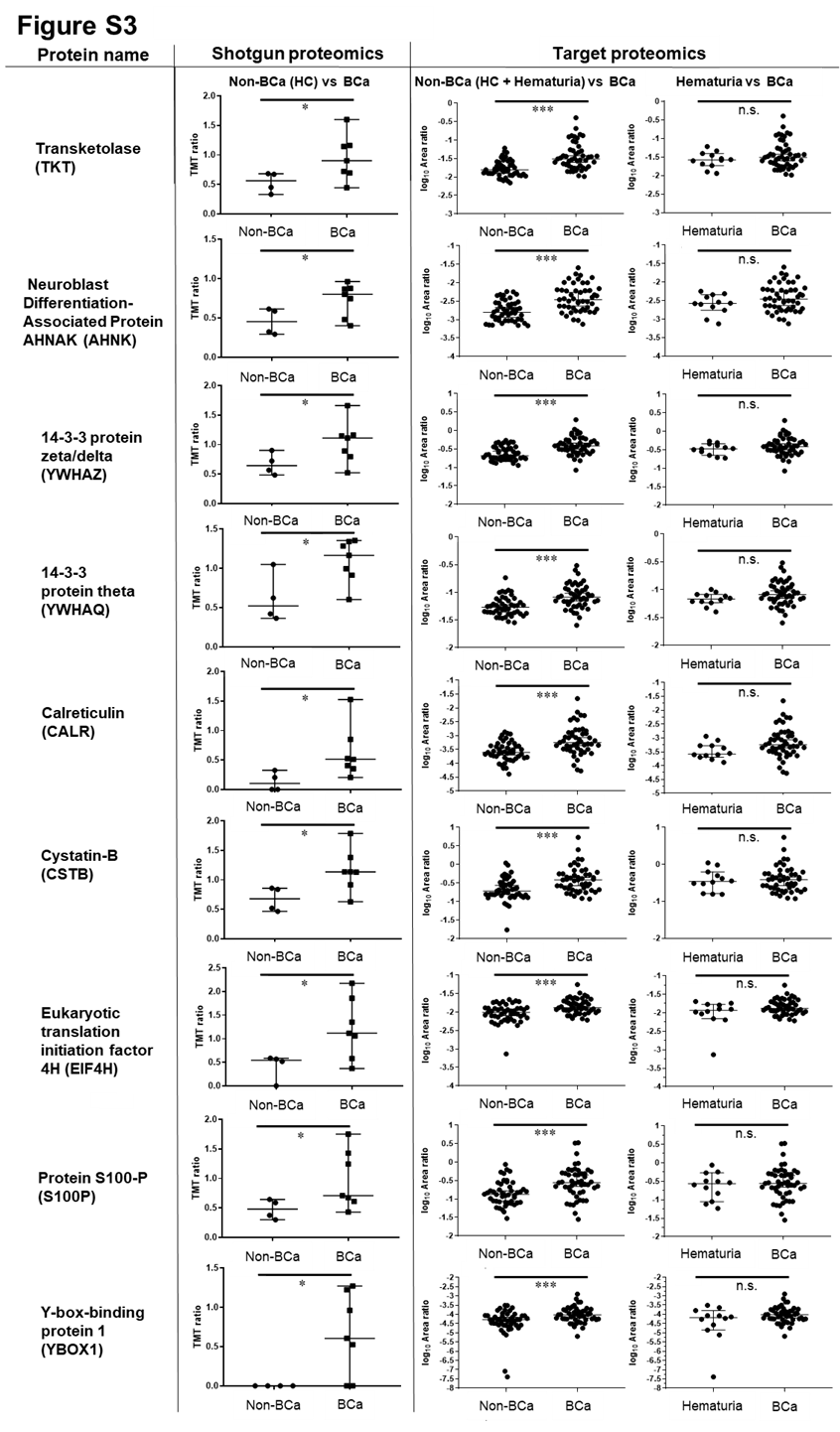
**

**Figure S3**

Relative quantification of nine uEV proteins between non-BCa patients (healthy controls: HC) and patients with BCa in shotgun proteomics (left), between non-BCa patients (HCs and hematuria patients) and BCa patients (center), and between hematuria and BCa patients (right) in targeted proteomics.

Comparisons were performed using Welch's t-test for shotgun proteomics and the Mann–Whitney U test for targeted proteomics (***p < 0.01, *p < 0.05, n.s.: not significant). Data are presented as mean with SD. uEV, urinary extracellular vesicles; BCa, bladder cancer.

**Figure S4**


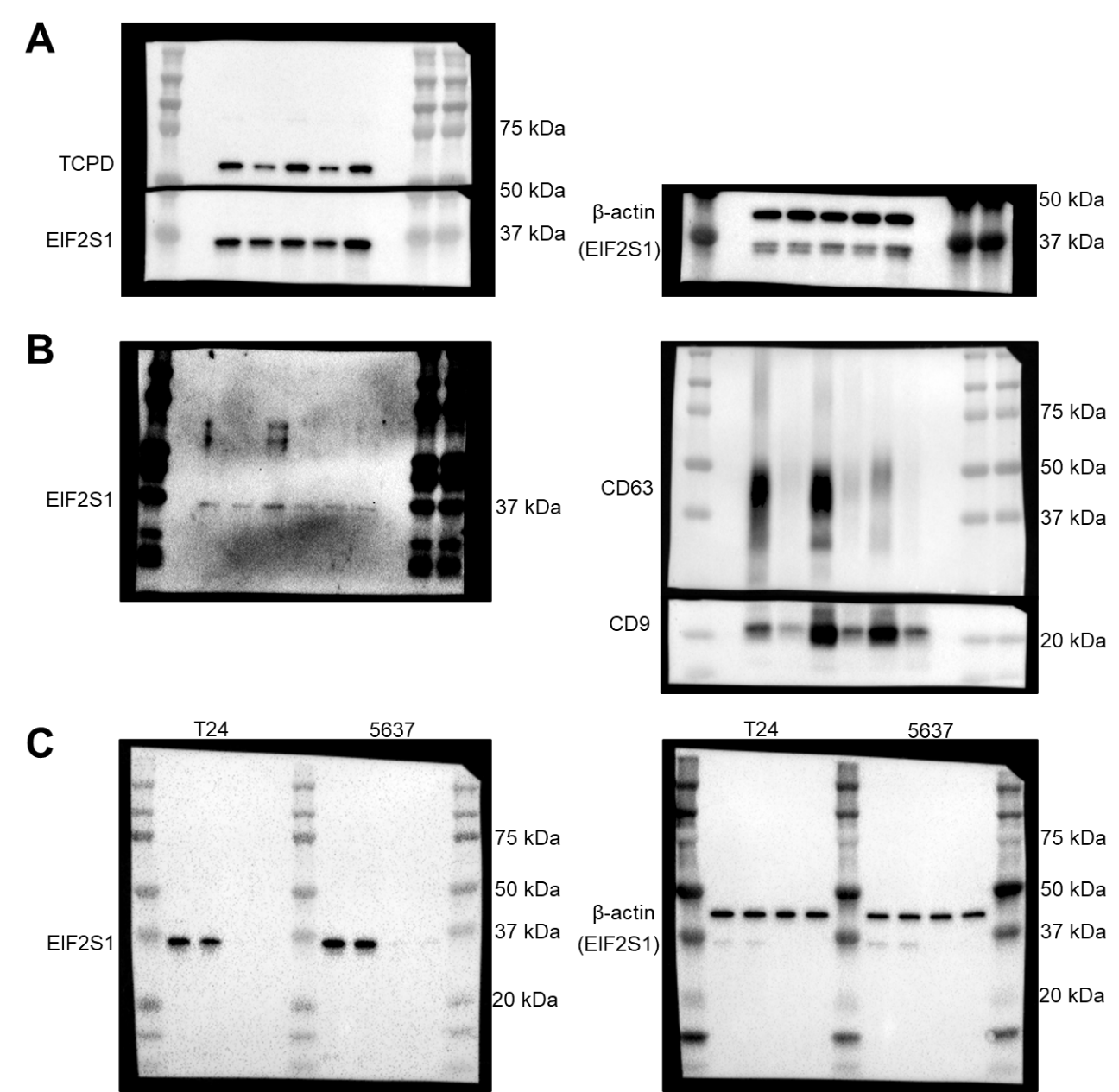


**Figure S4**

Uncropped western blot membranes corresponding to (A) Figure 2A, (B) Figure 2B, and (C) Figure 3A.

**Figure S5**


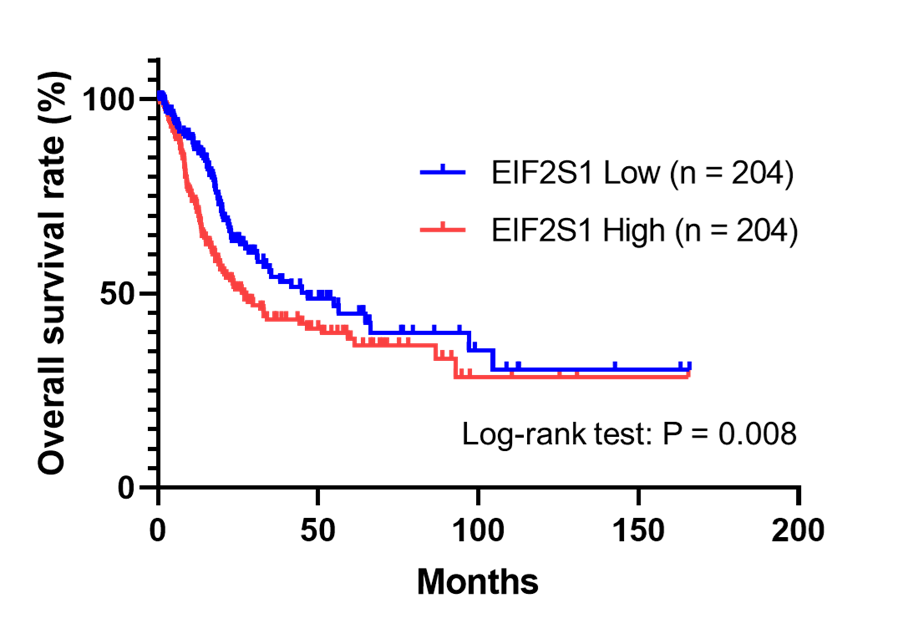


**Figure S5**

Kaplan–Meier analysis of overall survival comparing EIF2S1 expression low and high groups in the TCGA cohort (n = 408) (log-rank test).

**Figure S6**

**
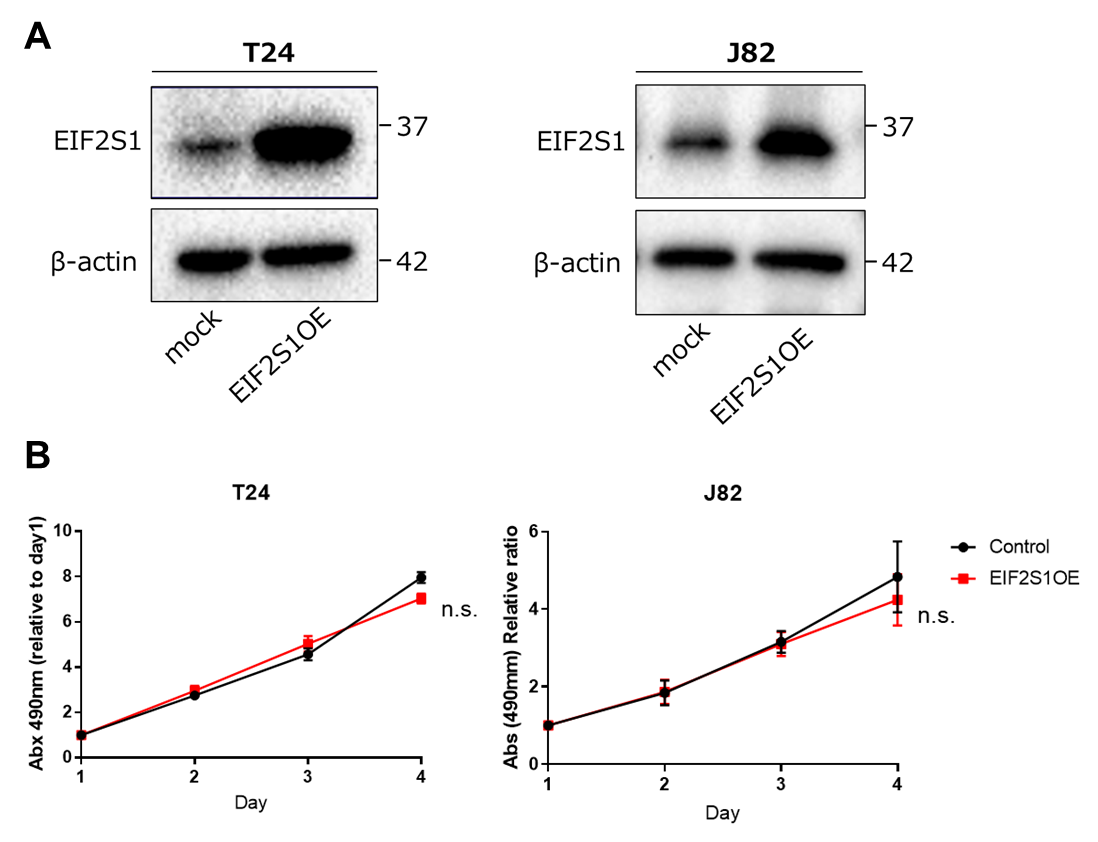
**

**Figure S6**

(A) Western blotting showing EIF2S1 overexpression (OE) in the lysates of T24 and J82 cells. (B) Stable EIF2S1-overexpressing (EIF2OE) and control cells were incubated for the indicated time periods (T24 and J82 cells). Cell proliferation was assessed using the MTS assay. Results are presented as mean with SD (n = 3) and analyzed using the Student’s t-test (***p < 0.01).
